# Understanding the meaning of the lived experience “maternal role” in women with multiple sclerosis and planning a supportive program: a combined exploratory study protocol

**DOI:** 10.1186/s12978-024-01799-w

**Published:** 2024-05-01

**Authors:** Elaheh Mansouri Ghezelhesari, Mohamad Ali Nahayati, Abbas Heydari, Hosein Ebrahimipour, Talat Khadivzadeh

**Affiliations:** 1 Student Research Committee, Mashhad University of Medical Science, Mashhad, Iran; 2 Department of Neurology, Ghaem Hospital, Mashhad University of Medical Sciences, Mashhad, Iran; 3 Nursing and Midwifery Care Research Center, Mashhad University of Medical Sciences, Mashhad, Iran; 4 Department of Health Economics and Management, School of Health, Mashhad University of Medical Sciences, Mashhad, Iran; 5 Nursing and Midwifery Care Research Center, Mashhad University of Medical Sciences, Ebn Sina Street, Mashhad, 9137913199 Iran; 6 Health Sciences Reseach Center, Torbat Heydarieh University of Medical Sciences, Torbat Heydarieh, Iran; 7 Department of Midwifery, School of Nursing and Midwifery, Mashhad University of Medical Sciences, Mashhad, Iran

**Keywords:** Multiple sclerosis, Maternal role, Program, Protocol

## Abstract

**Background:**

The maternal role is one of the most challenging yet rewarding roles that women experience in their lives. It begins when a woman becomes pregnant, and as the pregnancy progresses, she prepares to fulfill her role as a mother. A woman's health plays a crucial role in her ability to fulfill the maternal role. Multiple sclerosis (MS), as an autoimmune disease, presents unique challenges in achieving this role. Failing to fulfill the maternal role can have lasting consequences for both the mother and the baby. Given the increasing number of women with MS of reproductive age in Iran and the absence of specific programs for this group during pregnancy and postpartum, researchers have decided to develop a supportive program by exploring the meaning of the maternal role and identifying the needs of these women during this period.

**Methods/materials:**

This study will be conducted in 3 stages. The first stage involves a qualitative study to explore the meaning of the "maternal role" in women with MS through a descriptive and interpretive phenomenological approach based on Van Manen's method. Data will be collected through semi-structured interviews with pregnant women with MS and mothers with MS who have children under one-year-old, recruited from the Multiple Sclerosis Society of Mashhad, Iran. The second stage will involve designing a support program based on the findings of the phenomenological study, literature review, and exploratory interviews. A logical model will guide the development of the program, and validation will be conducted using the nominal group technique.

**Discussion:**

This study is the first of its kind in Iran to explore the meaning of the maternal role and develop a support program for women with MS. It is hoped that the results of this study will help address the challenges of motherhood faced by these women.

## Plain language summary

The maternal role is considered one of the most significant roles a woman will undertake in her lifetime. It is a process in which a woman, as a mother, attains competency in her role and eventually becomes comfortable with her identity as a mother. However, there are various factors, such as diseases, that can impede a mother from fully embracing her role. Multiple sclerosis (MS), an autoimmune disease that predominantly affects women of reproductive age, is one such condition.

Given the lack of research in Iran regarding the experiences of women with MS in their maternal role, a study was developed in three phases. The first phase involves interviewing pregnant women with MS and mothers with MS who have children under one-year-old to explore the meaning of the maternal role. In the second phase, utilizing the findings from the initial interviews and the experts' opinions, a support program will be created to assist women with MS during pregnancy and after giving birth, and in the last stage, this program will be evaluated by nominal group technique.

## Background

The maternal role is one of the most challenging yet rewarding roles that women experience in their lives [1]. This role is seen as a process in which a mother gains competence and ultimately feels comfortable with her identity as a mother [2]. It consists of two components: physical and practical duties such as feeding, holding, cleaning the baby, and ensuring its safety, as well as cognitive-emotional aspects focusing on attitudes towards motherhood and awareness of the child's needs [3].

Various factors influence a woman's ability to fulfill the maternal role [4], with health being a significant factor. Illness can diminish a woman's capacity and confidence in performing maternal duties, leading to delays in achieving the maternal role [5].

Multiple Sclerosis (MS), the third leading cause of neurological disability, is the most prevalent neurological disorder among young adults [6]. It is a chronic, progressive disease characterized by autoimmune damage to the brain, spinal cord, and optic nerve, resulting in a range of sensory, movement, and vision problems [7]. People suffering from this disease experience a wide range of sensory, movement, and vision problems due to extensive local inflammation in the central nervous system and disturbances in the transmission of nerve signals [8, 9].

According to the Multiple Sclerosis International Federation (MSIF) report, the number of MS patients worldwide has increased from 2.3 million people in 2013 to 2.9 million in 2023 [10]. However, the incidence of women is higher than men [11]. Therefore, MS is spreading more rapidly among women of reproductive age, women who are at the peak of their sexual and reproductive activities [12].

Having this disease causes a wide range of physical and mental disorders in women. In addition to physical symptoms such as urinary incontinence, bowel issues, menstrual irregularities, and sexual dysfunction, women with MS also experience psychological challenges like irritability, decreased self-esteem, anxiety, and depression [13–16].

MS can also affect women's fertility [17]. Before the 1950s, many affected women refused pregnancy because they were worried about the adverse effects of the disease on pregnancy [18]. However, advancements in disease treatment have made it possible for women with MS to have children [19]. Research shows that women with MS who have children experience slower disease progression compared to those who are single or childless [20–22]. Despite these positive outcomes, the number of women with MS who choose to become pregnant remains lower than the general population [23, 24].

Many affected women have attributed their low desire for pregnancy and motherhood to concerns that are influenced by the disease itself and its subsequent complications [25]. Damage to the fetus due to drug use during pregnancy, the risk of disease transmission to the fetus, and challenges related to caring for the baby are just some of the concerns these women face in regard to pregnancy and motherhood [18, 25–27].

Several studies related to the maternal role in affected women have reported that these women are not satisfied with their role as a mother. This lack of satisfaction with the role is caused, on one hand, by the inability of women to estimate the physical and psychological needs of their babies and children, and on the other hand, by the influence of guilt due to the possibility of disease transmission and neglecting the well-being of their babies and children in the future [25, 28]. They stated that this disease compromised their ability to perform some activities related to the "ideal mother," and in this case, they were judged as a "bad" or "abnormal" mother [18]. In fact, society viewed them as disabled mothers who did not have the necessary ability to care for their babies [27].

It appears that many affected women face challenges in achieving their role as a mother, and these challenges can impact the maternal interactions with children and the mother's understanding of the baby's future [29]. Failure to achieve this role leads to feelings of depression, stress, reduced maternal and child pleasure, delayed growth, child abuse, lack of emotional and social development in later stages, and ultimately a lack of necessary care [30–32].

Supporting and guiding women of reproductive age in fulfilling the maternal role is crucial to improving women's health [33]. The availability of support systems, both formal and informal, with a positive outlook, is necessary for successfully transitioning into the maternal role and building self-confidence in parenting during the prenatal and postnatal periods [31]. Many women with MS during pregnancy and after delivery express concerns about the lack of support from family and health personnel [25, 34]. Supporting these women can be considered a protective factor against the concerns and challenges caused by the disease's impact on children's well-being and maternal depression symptoms [35].

In women with MS, 3-6 months after giving birth, the most likely disease flare-up is due to the elimination of the immunosuppressive state of pregnancy and the sudden decrease in estrogen [18, 36]. At the same time, the physical and emotional support received from their families and parents decreases, intensifying the damage caused by the flare-up of the disease. Meanwhile, the social support received during this period plays a vital role in the transition to motherhood [25, 37].

In Iran, the prevalence of MS is very high. According to the MSIF report, the prevalence of this disease in Iran is 101 cases per 100,000 people [10]. In a study reported by Etemadifar et al., out of 42,200 MS patients registered in 2013 in Iran, 32,477 were women and 9,723 were men [38]. Despite the high prevalence of this disease in Iran, many quantitative studies have been conducted regarding the problems and challenges faced by women with MS [12, 39, 40], however, there are very few qualitative studies. This is despite the fact that in order to discover the lived experience of phenomena that somehow face human interactions, quantitative research does not have the necessary flexibility and depth [41]. Among the types of qualitative studies, phenomenology is a type of study that tries to provide a direct description of experiences, as they are, without considering their psychological basis and scientific and causal explanations [42]. By using phenomenology studies, it is possible to reveal the hidden meanings in people's experiences and, in this way, facilitate access to their complex and hidden needs by creating a common sense with others [43]. A perspective based on qualitative research in MS disease, focusing on the "voice of patients" and reflecting on their experiences, supports these people and their families during important life changes such as pregnancy and motherhood [44].

Considering the growing population of women of reproductive age with MS in Iran [45], and the lack of a qualitative study related to the experiences of these mothers from the role of motherhood and the challenges they face during pregnancy and after delivery, and the lack of guidelines or a comprehensive program resulting from their needs during this period, the present study was designed to discover the meaning of the maternal role in women with MS and design a support program based on their needs during pregnancy and the first year after childbirth.

### Study aim

The aim of this study is to gain a deep insight into the experiences of mothers in their maternal role. By doing so, we hope to design a support program that addresses the needs and challenges faced by these women during pregnancy and after childbirth. Ultimately, this will lead to an improvement in the quality of health services and prevent the adverse effects of not fulfilling the maternal role.

### Specific objectives

The primary objective of the first stage of the study is to conduct a phenomenological study to understand the significance of the maternal role in women with MS. This will help us identify the needs and challenges these women face during this time, which will inform the development of a support program in the second stage.

Special Objectives of the First Stage:

1. Explore the lived experience of the maternal role in pregnant women with MS and those who have a child less than one-year-old.
2. Identify the needs and expectations of mothers regarding a support program.

The overall goal of the second phase is to create a support program based on the findings of the phenomenological study, literature review, and interviews with key informants.

Special Objectives of the Second Stage:

1. Determine the needs and challenges for designing a support program based on the study results, literature review, and interviews.
2. Define the components, characteristics, and strategies of the support program using a logical model.

The general purpose of the third stage is to validate the support program using the nominal group technique.

Special Objectives of the Third Stage:

1. Validate the support program by gathering expert opinions during the nominal group meeting.
2. Formulate the final program based on feedback from experts in the nominal group meeting.

### The first stage

The initial phase of the current study utilizes Van Manen's descriptive and interpretive phenomenology method. This approach allows the researcher to explore a shared experience from various perspectives by interpreting the perceptions and lived experiences of different individuals [46]. Since the focus of this study is on understanding the subjective nature of the participants' experiences, both description and interpretation of the phenomenon are crucial. Van Manen's method, with its comprehensive explanation of the hermeneutic cycle and analysis, can aid in understanding the significance of the maternal role in women with MS.

#### Choosing the research field

To gather relevant information on the maternal role phenomenon, the researcher has selected health centers and the Multiple Sclerosis Society of Mashhad as the research field for this study.

#### Participant selection method

For maximum information, purposive (purposeful) sampling will be employed in this study. Participants will be chosen based on diversity in age, education, occupation, disease severity, duration of illness, and number of children. This approach aims to provide a comprehensive view of the maternal role phenomenon.

#### Inclusion criteria

Participants must have a confirmed MS diagnosis by a neurologist, be pregnant women with MS and have children under one year old, not have any other diseases besides MS, be able to understand and speak Persian, and communicate effectively with the researcher**.**

#### Exclusion criteria

Participants who are unwilling to continue cooperation at any stage of the interview will be excluded.

#### Sample size

The sampling process will conclude when no new data is obtained from interviews or when data saturation is reached.

#### Data collection process

After receiving approval from the ethics committee at Mashhad University of Medical Sciences, the researcher first contacts the Multiple Sclerosis Society and health centers. At this stage, eligible women who are willing to participate in the study sign an informed consent form and receive comprehensive explanations about the research objectives. Demographic information of the participants is then recorded to describe them. The participants are asked to choose a suitable place for the interview and are informed that a voice recorder will be used. They are assured that their voice will not be shared and their information will be coded on the demographic questionnaires.

During the interview, general questions are asked first, followed by specific questions related to the research problem. Initial questions include: "What made you decide that now is the right time to become a mother?" The main questions related to motherhood are then asked, such as "How do you perceive motherhood?" Probing questions like "Can you provide more details?" are used to gain a deeper understanding of the issue. Each interview typically lasts between 30 to 90 minutes, with an average of 45 minutes. Interviews continue until data saturation is reached. After conducting the interviews and recording the tapes, data interpretation begins. The interpretation process involves a spiral movement from part to whole and vice versa to ensure no information is overlooked.

#### Data analysis

Van Manen's third to sixth steps will be utilized to analyze the data. These steps involve reflecting on the intrinsic content of the phenomenon, writing and rewriting, maintaining a strong connection with the phenomenon, and matching the study's findings while considering the relationship between the parts and the whole. The Guba and Lincoln criteria will be applied to enhance the study's accuracy.

### Second stage

The second stage of the current study aims to design a support program for women with MS to address the challenges they face in fulfilling the maternal role. In this study, a logical model is utilized to create a support program that recognizes the needs and challenges of these women based on the findings of the phenomenological study. The use of a logical model has garnered significant attention among healthcare providers and is considered an appropriate model for planning and evaluating health issues in society [47].

A logic model is a transparent method that graphically illustrates the rationale behind an action. It is used to assess the effectiveness of designed programs and to outline the logic of implementing a program before its design. This model consists of various components, including Inputs, Activities/Strategies, Output, Assumptions, Short-term Outcomes, Medium-term Outcomes, and Impacts/Long-term Outcomes. [48].

The steps involved in developing a logical plan in this study include five steps, with the final step being completed in the third stage of the study. These steps are as follows:

1. Data collection: Data collection in this study to develop a support program based on a logical model through the results obtained from a qualitative study in the first stage, a systematic review of quantitative and qualitative studies conducted concerning the needs of women with MS in relationship with mother's role, with extensive search in scientific databases such as Pubmed, Scopus, EMBASE, Web of Science, The Cochran Library and Persian language databases such as SID and Magiran using Persian keywords and their English equivalents and specialized interviews with Key informants
2. Definition of the problem and statement of objectives: Based on the codes and themes extracted from the phenomenological study, exploratory interviews with key informants, and literature review, the needs and problems of women with MS regarding the maternal role will be identified to determine the goals of the support program.
3. Definition of program elements: In the logical model, the elements include inputs, activities, outputs, and short-term, medium-term, and long-term goals (consequences). In this model, the "if-then" set can explain the relationship between the components well. Ordering that "if" the resources (inputs) including... are provided, then the activities of... can be executed, or "if" the activities of the program are executed successfully, then the outputs of... .. and the consequences of ... will be obtained [49].

Identifying the required resources or inputs to respond to the needs and challenges of women with MS concerning the maternal role will be done based on literature review, exploratory interviews, and the use of professors' opinions.

Concerning the necessary activities and strategies, the question should be answered, what activities should be done on the designated resources to achieve the desired results? Identifying and describing the necessary activities and strategies for the implementation of the support program as in the previous stage using the results of the present phenomenological study, literature review, and exploratory interviews with key informants and experts, and finally consulting with the professors of the research team will be done. Short-term, medium-term, and long-term goals (consequences) will also be determined with the consensus of professors and the research team and exploratory interviews with experts.

4. Building and drawing the model: In this step, the relationship between the components of the logical model is specified.

### The third stage

In the final stage of evaluating the draft support program developed in this study, the nominal group technique will be employed. This technique involves four basic steps: generating ideas, recording ideas, discussing ideas further, and voting on ideas [50].

For the nominal group technique in this study, participants will be purposefully selected based on the research team's recommendations.

## Discussion

The pregnancy rate after a diagnosis of MS in women is lower than that of the normal population [23, 24]. However, several studies have suggested that pregnancy may help slow the progression of the disease [20–22]. Many affected women attribute their low desire for pregnancy and motherhood to concerns stemming from the disease itself and its complications. MS poses a significant challenge for women who wish to become pregnant and be mothers [25].

These challenges highlight the necessity of developing and implementing a program to address these needs and obstacles. Despite an extensive search, no comprehensive program or guidelines have been created for these mothers. Some studies have identified support as a crucial element during pregnancy and postpartum for these mothers [25, 37].

In Iran, the prevalence of MS is high [45]. In Iranian culture, which is influenced by the Islamic religion, having children is viewed as a social and cultural duty for women. Newly married women are expected to conceive shortly after starting married life. The role of mother is paramount for women in Iranian society, and they strive to fulfill this role correctly [51, 52]. However, comprehensive guidelines for these mothers during pregnancy and postpartum are lacking in Iran, with only limited references in national guidelines for pregnancy and childbirth care.

Due to the absence of phenomenological studies exploring the experiences of Iranian mothers with MS in their maternal role, and the lack of a comprehensive plan for them during pregnancy and postpartum, researchers have developed a support program based on the needs of these mothers. The aim is to reduce the challenges faced by these mothers and utilize qualitative data in practice. This study hopes to serve as an initial step for further research both within and outside of Iran.

## Data Availability

Not applicable.

## Abbreviations

MS: Multiple Sclerosis
MSIF: Multiple Sclerosis International Federation
